# Multi-omics characterization of blood metabolites and cervical microbiota associated with estrus in simmental cattle

**DOI:** 10.1186/s42523-026-00571-8

**Published:** 2026-04-15

**Authors:** Jiandong Wang, Xue Zhang, Youli Yu, Yanan Guo, Yuxi Zhao, Shijie Bi

**Affiliations:** 1 Institute of Animal Science, Ningxia Academy of Agricultural and Forestry Sciences, Yinchuan, Ningxia Hui Autonomous Region 750000 China; 2 College of Animal Science and Technology, Yunnan Agricultural University, Kunming, Yunnan Province 650201 China

**Keywords:** Simmental cattle, Estrus, Blood metabolites, Uterine microbiota

## Abstract

Accurate estrus detection is critical for optimizing reproductive efficiency in beef cattle. This study characterized blood metabolites and cervical microbiota associated with estrus in Simmental cattle using an integrated multi-omics approach. Twelve multiparous cows were assigned to estrus (*n* = 6) or nonestrus (*n* = 6) groups based on behavioral, hormonal, and ultrasonographic criteria. Serum samples were analyzed via ELISA and UPLC-MS/MS, while cervical microbiota were profiled using 16S rRNA and ITS sequencing. Serum biochemical analysis revealed elevated levels of total protein, albumin, glucose, and insulin-like growth factor-1 during estrus (*p* < 0.01). Untargeted metabolomics identified 258 differentially abundant metabolites enriched in amino acid and lipid metabolism pathways. Cervical microbiota analysis showed that *Firmicutes* and *Bacteroidetes* were the dominant bacterial phyla, with *Bacteroidetes* increasing by 13.36% during estrus. Fungal *Ascomycota* dominated the estrus group (78.38% vs. 67.20%), accompanied by reduced alpha diversity (*p* < 0.05). LEfSe analysis identified 22 bacterial and 16 fungal biomarkers associated with estrus. Correlation analysis revealed that E2 was positively correlated with *Bacteroidota* (*r* = 0.604) and fungal genera *Bacteroides* (*r* = 0.703), while progesterone showed negative correlations. This study reveals a complex microbiota-metabolite-host network specific to estrus in Simmental cattle, providing potential biomarkers for estrus detection.

**Graphical Abstract:**

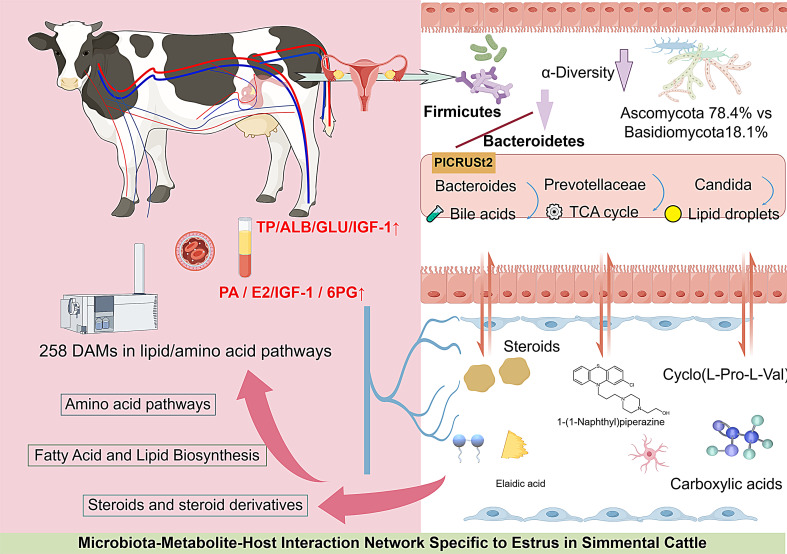

**Supplementary Information:**

The online version contains supplementary material available at 10.1186/s42523-026-00571-8.

## Highlights

First, multiomics profiling of blood metabolites and the cervical microbiota/fungi in Simmental cattle during estrus revealed estrus-specific signatures.

Estrus-associated fungal shift: dominance of *Ascomycota* (78.38%) with reduced diversity, linked to glycogen metabolism (*Saccharomycopsis*) and potential associations with estrogenic pathways (LEfSe LDA > 4).

Key metabolic pathways: A total of 258 differentially abundant metabolites enriched in amino acid (tryptophan, arginine) and lipid metabolism (sphingolipids) pathways were correlated with *Bacteroidetes* enrichment (+ 13.36%).

Practical biomarkers: Serum TP/ALB/GLU levels and Prevotellaceae/Ruminococcaceae abundance as potential indicators for estrus detection in beef cattle.

Methodological rigor: Integrated UPLC-MS/MS metabolomics, 16S/ITS sequencing, and LEfSe analysis provide a template for studying host‒microbe interactions in livestock.

**Supplementary Information:**

The online version contains supplementary material available at 10.1186/s42523-026-00571-8.

## Introduction

The beef cattle industry holds a significant position in China’s agriculture. China’s beef cattle industry is moving toward ecological, large-scale, and intensive development, but low reproductive efficiency remains a key bottleneck hindering industrial upgrading [1]. The continuous decline in the number of breeding cows, the generally low conception rate, and the poor survival rate of calves have severely impacted the economic efficiency of beef cattle farming. Against this backdrop, an in-depth exploration of the reproductive physiological mechanisms of cows is highly important for enhancing the competitiveness of the beef cattle industry [2, 3]. In research on the reproductive physiology of beef cattle, analyzing the physiological characteristics of the estrus cycle is highly important for improving reproductive efficiency. Existing evidence indicates that the intensity of estrus expression is significantly positively correlated with fertility [4, 5]. This correlation may stem from the specific physiological state during estrus [5]. Estrus, a critical physiological stage in the reproductive cycle of beef cattle, directly influences reproductive efficiency through dynamic changes in blood metabolic characteristics and the uterine microbial composition [6].

The cervical microbiota of cattle plays a crucial role in bovine reproductive health. In recent years, researchers have employed high-throughput sequencing technologies to uncover key aspects of microbial ecology within the bovine uterus. Studies indicate that bacterial diversity is essential for maintaining uterine health. However, there is currently no consensus on the exact composition of a healthy cervical microbial community. Typically, loss of bacterial diversity (or dysbiosis) can lead to uterine infections and is associated with increased relative abundances of bacteria such as *Bacteroides*, *Fusobacterium*, *Trueperella*, and *Porphyromonas* [7]. To gain deeper insights into the ecology of the bovine cervical microbiota, researchers have also investigated integrated analyses of the cervical microbiome and metabolome. Through such multiomics approaches, scientists have identified disease-associated metabolites that may provide competitive advantages to major uterine pathogens. These metabolites could serve as targets for future interventions aimed at suppressing the proliferation of opportunistic pathogens in the uterus [8]. Emerging evidence suggests that host genetic background influences reproductive tract microbiota composition, with significant differences observed between cattle breeds [9, 10]. The reproductive tract microbiome plays an important role in bovine fertility and overall health [11]. However, breed-specific microbial dynamics during estrus remain largely unexplored, particularly in Simmental cattle. Consequently, an in-depth understanding of the cervical microbiota and its interactions with the host is essential for developing strategies to prevent uterine disorders and improve reproductive management.

Moreover, metabolomics studies have provided novel perspectives for understanding estrus regulation. Metabolic research during the estrus phase constitutes a critical component in comprehending the reproductive physiology of beef cattle [10, 12]. Furthermore, metabolic studies in dairy cows have revealed significant differences in serum metabolites between cows with inactive ovaries and those exhibiting normal estrus cycles [13]. However, research on metabolic dynamics during the estrus cycle in beef cattle remains relatively scarce.

This study systematically investigated Simmental cattle through integrated multiomics approaches, employing an enzyme-linked immunosorbent assay to analyze serum biochemical parameters and hormone levels, ultraperformance liquid chromatography‒tandem mass spectrometry to characterize blood metabolic profiles, and 16 S rRNA/internal transcribed spacer sequencing to characterize cervical microbiota dynamics. The aim was to characterize blood metabolites and cervical microbiota associated with estrus in Simmental cattle. By integrating metabolomic and microbiomic data, this study describes host-microbe-metabolic interactions during estrus, focusing on amino acid/lipid metabolism and cervical bacterial/fungal communities. These findings provide descriptive data on estrus-associated changes in Simmental cattle and may inform future research on reproductive physiology and estrus detection.

## Materials and methods

### Experimental animals and design

This study employed a cross-sectional design. Twelve multiparous Simmental cows (parity = 2; age: 4.0–4.5 years) with uniform body condition scores (BCS 3.0–3.4; Supplementary Table 1) were assigned to either an estrus group (*n* = 6) or a nonestrus group (*n* = 6) based on their reproductive status at the time of sampling. Each animal was sampled once—blood and cervical swabs were collected at a single time point.

The estrus group consisted of cows exhibiting characteristic estrus behavior (scores 2–3; Supplementary Table 2), including standing heat and clear mucus discharge, along with fern-like vaginal mucus crystallization and low PROG levels (< 1 ng/mL). Ultrasonography confirmed the presence of dominant follicles (8.5–11.0 mm; Supplementary Table 3) in this group. In contrast, the nonestrus group showed a complete absence of estrus signs (score 0; Supplementary Table 2), elevated PROG concentrations (> 2.8 ng/mL), and the presence of corpora lutea on ultrasound (Supplementary Table 3), confirming their diestrus status. The physiological status of nonestrus cows was further validated by hormonal profiles and ovarian morphology, effectively excluding anestrus or cystic ovarian conditions.

### Sample collection

Blood was aseptically collected from the coccygeal vein approximately 10 cm from the tail base. The samples were subsequently centrifuged at 3,000 × g for 15 min, after which the serum aliquots (2.5 mL) were stored at -20 °C until analysis.

The cows were restrained in the standing position for cervical sampling. After fecal evacuation and vulva disinfection, sterile extended swabs (15 cm) were inserted into the vaginal canal to reach the cervical os. The swabs were rotated gently against the cervical surface for 10 s to collect mucosal secretions, then immediately placed in cryovials and flash-frozen in liquid nitrogen. All the samples were ultimately stored at -80 °C.

### Serum biochemical index measurement

An enzyme-linked immunosorbent assay (ELISA) was used to measure the serum levels of total protein (TP), albumin (ALB), globulin (GLO), the albumin-to-globulin ratio (A/G), cholesterol (CH), and glucose (GLU) in the experimental cows. The intra-assay and inter-assay coefficients of variation (CVs) were < 10% and < 15%, respectively, for all assays. All ELISA kits were obtained from Enzyme-linked Biotechnology Co., Ltd. (Wuhan, China). All samples were analyzed in duplicate.

### Serum hormone level measurement

ELISA was used to determine the serum levels of estradiol (E2), progesterone (PROG), inhibin-B (INH-B), and insulin-like growth factor-1 (IGF-1) in the experimental cows. The intra-assay and inter-assay coefficients of variation (CVs) were < 10% and < 15%, respectively, for all ELISA assays. Spectrophotometry was used to measure the serum levels of pyruvic acid (PA) and glucose-6-phosphate (6PG). The intra-assay and inter-assay CVs for PA and 6PG were < 5% and < 8%, respectively. All samples were analyzed in duplicate.

### UPLC‒MS/MS analysis

Serum samples (100 µL) were mixed with 400 µL of cold methanol/acetonitrile (1:1, v/v), sonicated for 1 h, incubated at − 20 °C for 1 h, and centrifuged at 14,000 × g for 20 min at 4 °C. The supernatant was vacuum-dried and reconstituted for LC‒MS analysis.

Chromatographic separation was performed on an ACQUITY UPLC HSS T3 column (2.1 × 100 mm, 1.8 μm; Waters) using a Shimadzu Nexera X2 LC-30AD system. Mobile phase A was 0.1% formic acid in water, and mobile phase B was acetonitrile. The gradient was: 0% B (0–2 min), 0–48% B (2–6 min), 48–100% B (6–10 min), 100% B (10–12 min), then re-equilibrated at 0% B for 3 min. Flow rate was 0.3 mL/min, column temperature 40 °C, and injection volume 4 µL.

Mass spectrometry was performed on a Q Exactive Plus (Thermo Scientific) in both positive and negative ESI modes. Source parameters: spray voltage ± 3.8/3.2 kV; capillary temperature 320 °C; sheath gas 30 arb; auxiliary gas 5 arb; probe heater 350 °C; S-lens RF level 50. Full scans (m/z 70–1050) were acquired at 70,000 resolution, and MS/MS scans at 17,500 resolution. Maximum injection times were 100 ms (MS) and 50 ms (MS/MS), with stepped normalized collision energies of 20, 30, and 40.

For quality control, a pooled QC sample was prepared by mixing equal aliquots of all serum samples and injected at regular intervals throughout the analytical run to monitor instrument stability. Relative quantification was performed using total peak area normalization; internal standards were not used in this untargeted metabolomics analysis.

### Cervical microbiome profiling in beef cattle

Genomic DNA was extracted from cervical swabs using the FastPure Microbiome DNA Isolation Kit (Vazyme, Nanjing, China) following the manufacturer’s instructions. DNA quality was assessed by 1.2% agarose gel electrophoresis and Nanodrop spectrophotometry, and used as a PCR template. The V3–V4 region of the 16 S rRNA gene (primers: 341 F: 5′-ACTCCTACGGGAGGCAGCA-3′; 806R: 5′-GGACTACHVGGGTWTCTAAT-3′) and the ITS1 region (primers: ITS1F: 5′-GGAAGTAAAGTCGTAACAAGG-3′; ITS2R: 5′-GCTGCGTTCTTCATCGATGC-3′) were amplified. PCR products were quantified (Quant-iT PicoGreen dsDNA Assay Kit; BioTek FLx800 microplate reader), and libraries were prepared (TruSeq Nano DNA LT Kit, Illumina). After size selection (2% agarose gel), paired-end sequencing was performed on an Illumina MiSeq platform.

Raw sequencing data were processed using QIIME2 (version 2019.4; https://qiime2.org/) [14]. Briefly, primers were trimmed using the cutadapt plugin [15], and sequences were then quality-filtered, denoised, merged, and chimera-removed using the DADA2 plugin [16] to generate amplicon sequence variants (ASVs). Singleton ASVs were removed. Taxonomy assignment was performed using a pre-trained Naive Bayes classifier against the SILVA database (Release 132; https://www.arb-silva.de/) [17] for 16 S data and the UNITE database (Release 8.0; https://unite.ut.ee/) [18] for ITS data. ASV tables were rarefied to the minimum sequencing depth (95% of the lowest sample depth) prior to diversity analyses.

### Statistical methods

Metabolomics data: Raw data were processed using MS-DIAL software (version 4.9; http://prime.psc.riken.jp/compms/msdial/main.html) [19] for peak alignment, retention time correction, and area extraction. Metabolites were identified by exact mass (< 10 ppm) and MS/MS matching against the HMDB database (version 5.0; https://hmdb.ca/) [20], MassBank (https://massbank.eu/) [21], GNPS (https://gnps.ucsd.edu/) [22], and an in-house database (BP-DB). PCA, PLS-DA, and OPLS-DA were performed using SIMCA software (version 14.1; Umetrics, Umeå, Sweden) with permutation tests (*n* = 200) for model validation. Differentially abundant metabolites were identified using Welch’s t-test followed by false discovery rate (FDR) correction (Benjamini–Hochberg). Metabolites with FDR-adjusted q < 0.05 and |log2FC| ≥ 1 were considered significantly altered. Hierarchical clustering and heatmaps were generated using R software (version 4.5.1; https://www.r-project.org/) [23] with the pheatmap package. Pathway enrichment analysis was performed using the KEGG database (https://www.genome.jp/kegg/) [24] and MetaboAnalyst (version 5.0; https://www.metaboanalyst.ca/) [25].

Microbiome data: Alpha diversity indices (Chao1, observed species, Shannon, Simpson) were compared between groups using Welch’s t-test. Beta diversity was assessed by principal coordinate analysis (PCoA) based on Bray–Curtis dissimilarity, with significance tested by PERMANOVA (999 permutations). Differential taxa were identified using LEfSe [26] (LDA score > 2.0, FDR-adjusted q < 0.05). Functional profiles of bacterial communities were predicted using PICRUSt2 (version 2.5.1) [27], and fungal functions using FUNGuild [28]. These predictions represent potential metabolic capacities and should be interpreted as hypothesis-generating.

Correlation analysis: Correlations between microbial taxa (at genus level) and significantly altered metabolites were assessed using Spearman’s rank correlation, with FDR correction applied. Correlations with q < 0.05 and |r| > 0.6 were considered significant and are presented as exploratory findings. Correlation networks were visualized using R software (version 4.5.1) with the igraph and ggraph packages.

## Results

### Blood biochemical and hormonal characteristics of estrus in simmental cows

**Fig. 1:**
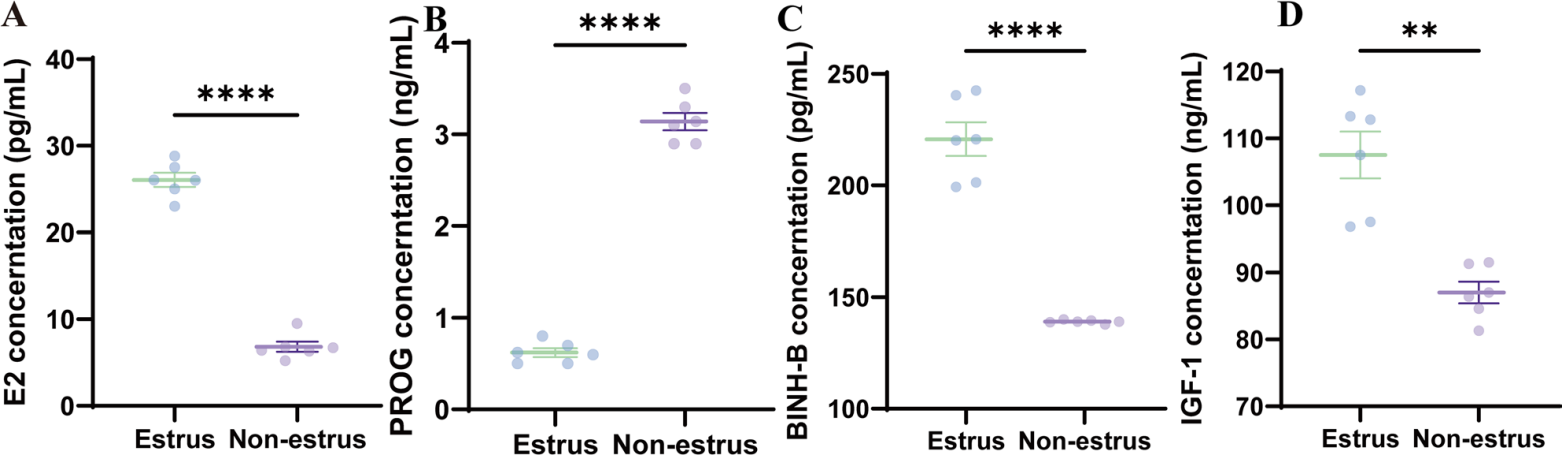
Comparative analysis of reproductive hormones and growth factors. (**A**) E2. (**B**) PROG. (**C**) INH-B. (**D**) IGF-1. Data represent the mean ± SEM (*n* = 6 per group; Welch’s t test, **p < 0.01, ****p < 0.0001)

To assess hormonal changes, the serum concentrations of E2, PROG, INH-B, and IGF-1were analyzed. Compared with nonestrus cattle, estrus cattle presented significantly elevated E2, INH-B, and IGF-1 levels (*p* < 0.01) (Fig. 1A, C&D), whereas PROG concentrations were significantly lower during estrus (*p* < 0.01) (Fig. 1B).

**Fig. 2:**
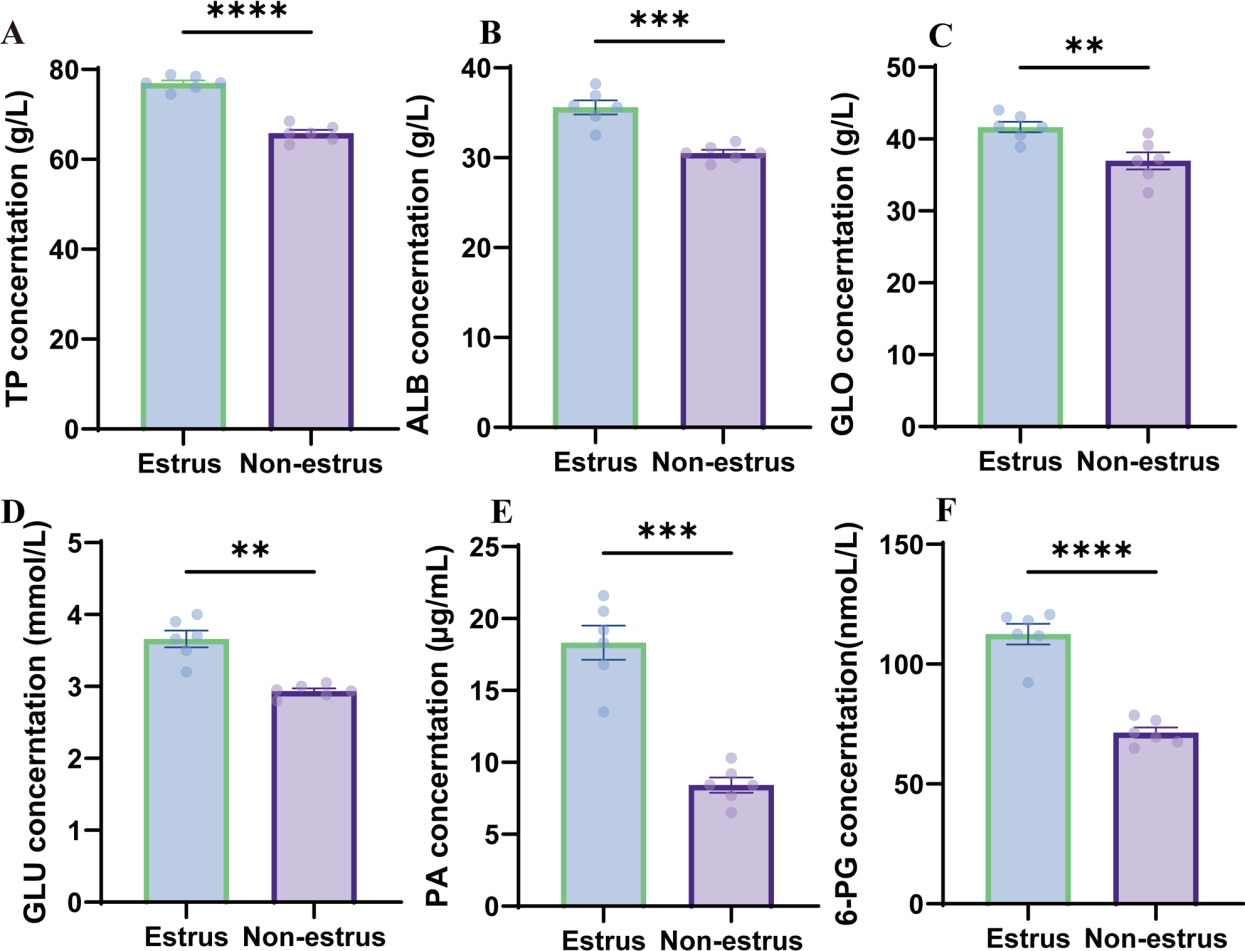
Blood biochemical profiles during estrus in cattle. (**A**) TP. (**B**) ALB. (**C**) GLO. (**D**) GLU. (**E**) PA. (**F**) 6PG content. Data represent the mean ± SEM (*n* = 6 per group; Welch’s t test, *p < 0.05, **p < 0.01, ***p < 0.001, ****p < 0.0001)

To investigate the effects of estrus status on blood biochemical parameters, the serum levels of TP, ALB, GLO, and GLU were measured via ELISA kits. The results revealed that the serum TP, ALB, and GLU concentrations were significantly greater (*p* < 0.01) in estrus cows, whereas the GLO levels were moderately greater (*p* < 0.05), indicating enhanced protein and glucose metabolism during estrus (Fig. 2A-F). Metabolic indicators, suggesting PA and 6PG, moderately increased (*p* < 0.01) during the estrus phase.

### Blood metabolic profiles of estrus in simmental cows

**Fig. 3:**
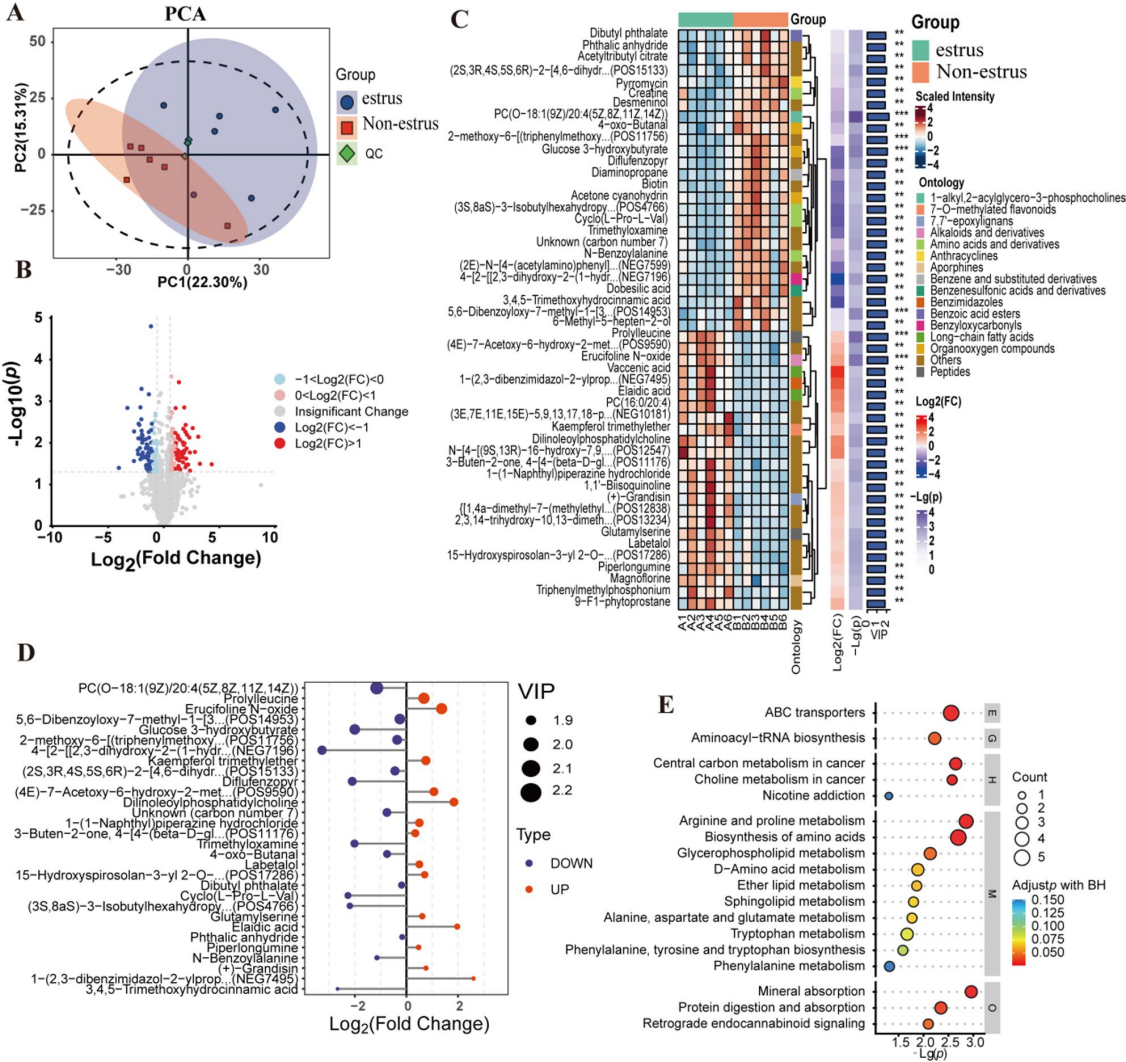
Serum metabolomic analysis of estrus vs. nonestrus cattle. (**A**) PCA score plot. (**B**) Volcano plot of differentially abundant metabolites. (**C**) Heatmap of group (**D**) VIP top 30 metabolites with fold changes. (**E**) KEGG pathway enrichment bubble plot (**E**: environmental information processing, **G**: genetic information processing, **M**: metabolism, **H**: human diseases, **O**: organismal systems)

To explore metabolic alterations during estrus, UPLC‒tandem mass spectrometry (UPLC‒MS‒MS/MS) was used to compare the serum metabolomes of estrus and non-estrus cows. PCA and PLS-DA revealed clear separation between groups (Fig. 3A & Supplementary Fig. 1B). Permutation tests of the PLS-DA model confirmed reliability (all permuted Q² values < original Q²), indicating no overfitting (Supplementary Fig. 1A). Using thresholds of |log2FC| ≥ 1 and FDR-adjusted q < 0.05, a total of 258 differentially abundant metabolites (DAMs) were identified between the estrus and nonestrus groups, comprising 128 upregulated and 130 downregulated metabolites in the estrus group (Fig. 3B).

Compared with non-estrus cattle, the estrus group presented decreased serum levels of amino acids and derivatives (mainly Cycl (L-Pro-L-Val) and N-Benzoylalanine) and organooxygen compounds (mainly Diflufenzopyr and Acetone cyanohydrin), whereas long-chain fatty acids and peptides were increased (Fig. 3C). Further classification revealed that the differentially abundant metabolites were primarily categorized as fatty acyls (40, 16.67%), benzene and substituted derivatives (24, 10%), and carboxylic acids and derivatives (26, 10.83%) (Supplementary Fig. 1C). The screening of the top 30 VIP metabolites revealed that PC(0–18:1(9Z)/20:4(5Z,8Z,11Z,14Z)) was significantly downregulated during estrus, whereas the levels of prolylleucine and erucifoline N-oxide were significantly upregulated (Fig. 3D). KEGG enrichment analysis revealed that the differentially abundant metabolites were involved mainly in ABC transporters, aminoacyl-tRNA biosynthesis, arginine and proline metabolism, and amino acid biosynthesis (Fig. 3E). Characteristic metabolite analysis revealed that PC(0–18:1(9Z)/20:4(5Z,8Z,11Z,14Z)), 6-hydroxypseudooxynicotine, and erucifoline N-oxide were the most significant metabolites (Supplementary Fig. 1D). In addition, among the 258 differentially abundant metabolites these metabolites represent potential biomarkers for estrus detection in cattle.

### Cervical bacterial microbiota characteristics of estrus in simmental cows

**Fig. 4:**
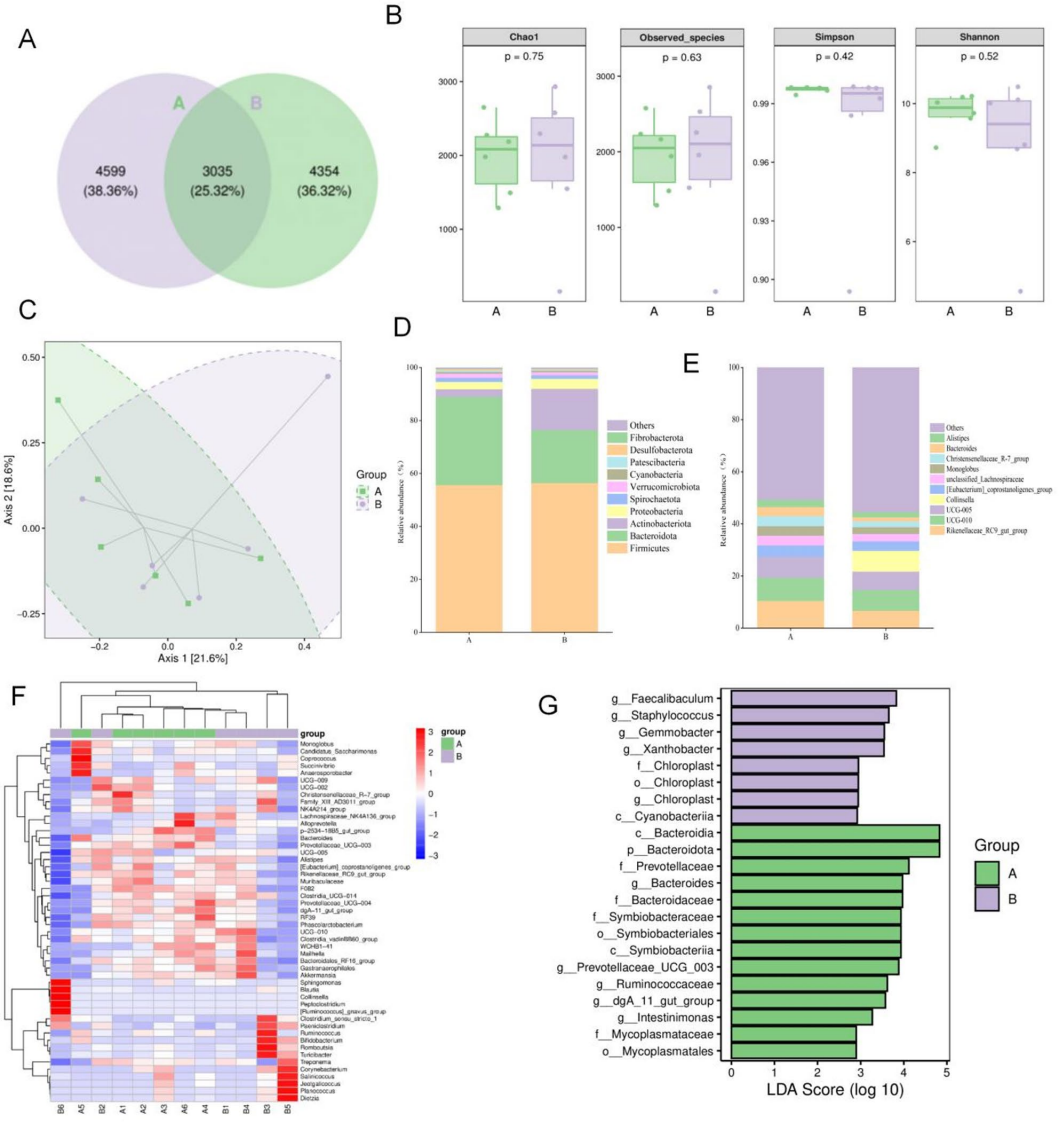
Bacterial community structure in the cervical microbiota. (**A**) OTU distribution. (**B**) α diversity indices. (**C**) PCoA clustering. (**D**) Dominant phyla (*Firmicutes*, *Bacteroidetes*). (**E**) Genus-level composition. (**F**) Clustered heatmap of the dominant genera. (**G**) LEfSe-identified bacterial biomarkers (LDA > 2). Data from 16 S rRNA sequencing (*n* = 6/group)

In this study, 12 cervical swab samples (6 estrus (A) and 6 nonestrus (B) cows) were collected. After 16 S rRNA gene sequencing, 1,336,059 raw reads were obtained, with 1,148,942 high-quality sequences retained after quality control. A total of 11,988 operational taxonomic units (OTUs) were identified, with 7,634 (A) and 7,389 (B) unique OTUs per group and 3,035 shared OTUs (25.32%) (Fig. 4A). Alpha diversity analysis showed that estrus cows exhibited lower Chao1 and observed species indices, indicating reduced microbial richness; the Shannon and Simpson indices were also decreased, suggesting lower overall diversity and altered community evenness (Fig. 4B). Beta diversity analysis (PCoA) revealed clustering within groups, suggesting similar cervical microbiota structures between A and B (Fig. 4C). At the phylum level, *Firmicutes* (55.60%) and *Bacteroidetes* (33.29%) dominated A, whereas *Firmicutes* (56.42%), *Bacteroidetes* (19.93%), and *Actinobacteria* were predominant in B. Compared with B, A presented a 0.82% decrease in *Firmicutes* and a 13.36% increase in *Bacteroidetes* (Fig. 4D). At the genus level, *Rikenellaceae_RC9_gut_group*, UCG-010, and UCG-005 were dominant in both groups, with *Collinsella* being additionally abundant in B (Fig. 4E). Heatmap analysis of the 50 most abundant genera revealed significant differences in the cervical microbiota composition between the estrus and nonestrus states. The estrus group presented increased relative abundances of *Christensenellaceae R-7_group*, *Alloprevotella*, and *Phascolarctobacterium*. In contrast, the nonestrus group was characterized by relatively high abundances of *Clostridium sensu stricto 1*, *Treponema*, and *Corynebacterium*. Notably, *Bifidobacterium* was detected in only the nonestrus samples (Fig. 4F). LEfSe analysis identified 22 differential bacterial biomarkers (LDA score > 2): A (Estrus): *Bacteroidia*, *Bacteroidota*, *Prevotellaceae*, *Bacteroides*, *Bacteroidaceae*,* Symbiobacteraceae*, *Symbiobacteriales*, *Symbiobacteriia*, *Ruminococcaceae*,* Intestinimonas*, *Mycoplasmataceae*, *and Mycoplasmatales.* B (nonestrus): *Faecalibaculum*, *Staphylococcus*, *Gemmobacter*, *and Xanthobacter* (Fig. 4G).

These results identify distinct bacterial taxa associated with estrus status. The estrus group was characterized by increased relative abundances of *Prevotellaceae*, *Bacteroidaceae*, and *Ruminococcaceae*, while the nonestrus group showed enrichment of *Faecalibaculum*, *Staphylococcus*, *Gemmobacter*, and *Xanthobacter*. These taxa represent potential microbial signatures of reproductive status in Simmental cattle.

### Cervical fungal microbiota characteristics of estrus in simmental cows

**Fig. 5:**
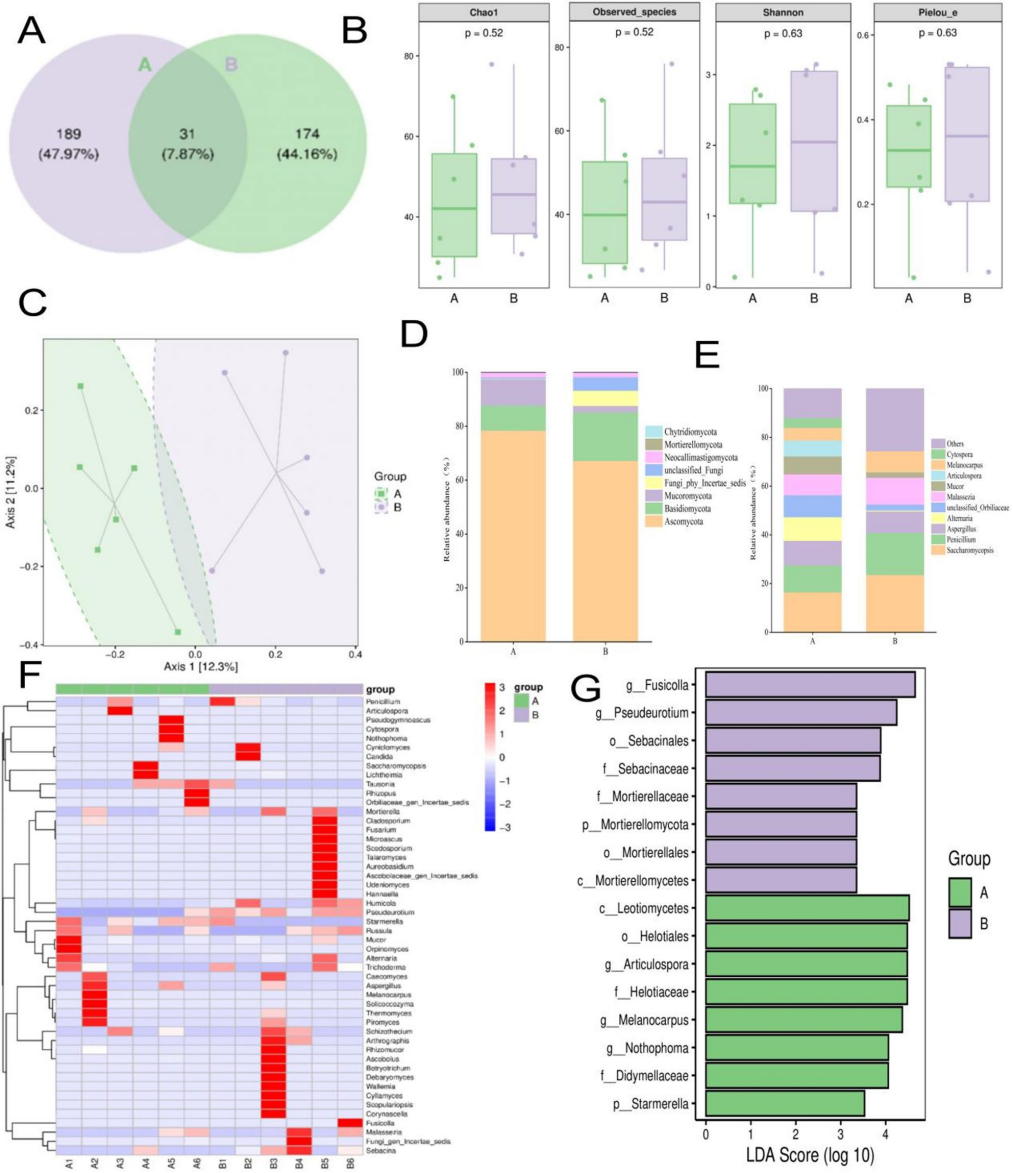
Fungal community structure in the cervical microbiota. (**A**) OTU distribution. (**B**) α diversity indices. (**C**) PCoA clustering. (**D**) Dominant phyla (*Ascomycota*, *Basidiomycota*). (**E**) Genus-level composition. (**F**) Clustered heatmap of the dominant genera. (**G**) LEfSe-identified fungal biomarkers (LDA > 2). Data from ITS sequencing (*n* = 6/group)

ITS sequencing of 12 cervical swabs yielded 1,377,264 raw reads (1,140,588 high-quality sequences). A total of 394 OTUs were identified, with 189 (A) and 174 (B) unique OTUs and 31 shared OTUs (7.87%) (Fig. 5A). Estrus was associated with significantly lower fungal alpha diversity (Chao1, observed species; Shannon, Simpson; Fig. 5B). Beta diversity analysis (PCoA) revealed clear separation between groups (Fig. 5C). At the phylum level, *Ascomycota* dominated A (78.38%) and B (67.20%), with *Basidiomycota* (18.08%) notably present in B (Fig. 5D). Genus-level analysis revealed *Saccharomycopsis* as dominant in A, whereas B presented *Saccharomycopsis* and *Penicillium* (Fig. 5E). The heatmap revealed distinct distribution patterns of key fungal genera: *Pseudeurotium* was consistently more abundant in the nonestrus samples, whereas *Starmerella* was more prevalent in the estrus samples (Fig. 5F). LEfSe identified 16 fungal biomarkers: A (Estrus): *Leotiomycetes*, *Helotiales*, *Articulospora*, *Helotiaceae*, *Melanocarpus*, *Nothophoma*, *Mortierellomycota Didymellaceae*, *and Starmerella. B (nonestrus): Fusicolla*,* Pseudeurotium*, *Sebacinales*, *Sebacinaceae*, *Mortierellaceae*, *Mortierellomycota*, *Mortierellales*, *and Mortierellomycetes* (Fig. 5G).

These results reveal fungal community shifts associated with estrus. The estrus group exhibited higher relative abundances of *Ascomycota* (particularly *Saccharomycopsis*, *Starmerella*, and *Articulospora*), whereas the nonestrus group showed enrichment of *Mortierellomycota* and P*seudeurotium*. These taxa may serve as fungal markers of reproductive status in cattle.

### Microbial functional shifts and blood metabolite correlations with estrus status

**Fig. 6:**
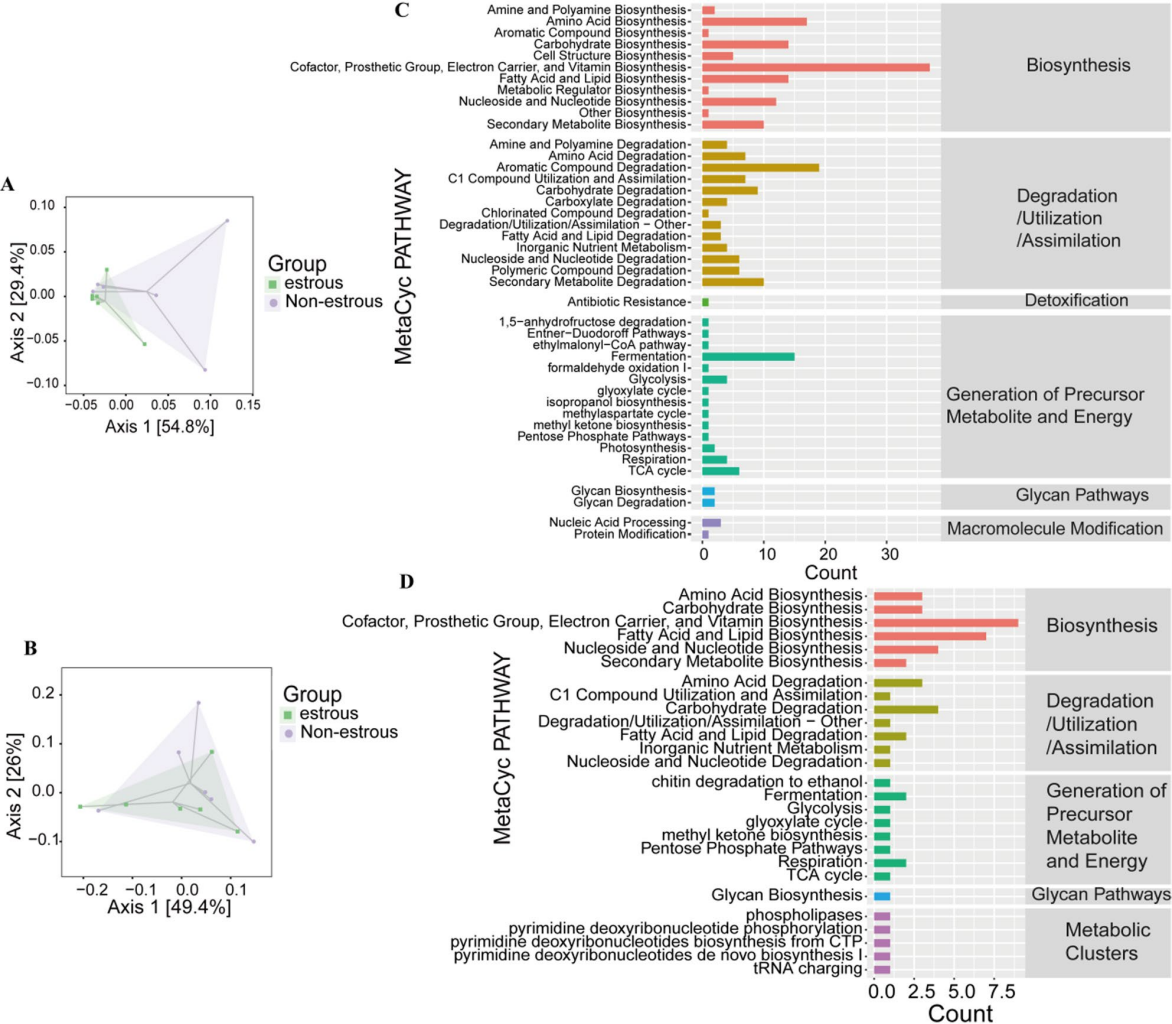
Functional prediction and metabolite correlation analysis. (**A**) PCoA of bacterial functional profiles (PICRUSt2) showing clustering patterns. (**B**) PCoA of fungal functional profiles. (**C**) MetaCyc pathway abundance of bacterial communities, highlighting key metabolic pathways. (**D**) MetaCyc pathway abundance of fungal communities

The analysis revealed significant differences in microbial metabolic profiles between the estrus and nonestrus states (Fig. 6A). Principal coordinate analysis clearly revealed separation of the bacterial functional profiles between the groups (Fig. 6B; PERMANOVA *P* = 0.003), with estrous-associated bacteria enriched in the Cofactor, Prosthetic, and Electron Carrier groups, as did the Vitamin Biosynthesis, Aromatic Compound degradation, and Fermentation groups (Fig. 6 C). Fungal communities displayed distinct clustering patterns (Fig. 5D), with estrous-associated fungi demonstrating enrichment in the Cofactor, Prosthetic, and Electron Carrier Groups and in the Vitamin Biosynthesis, Fatty Acid and Lipid Biosynthesis, Carbohydrate Degradation, Fermentation and Respiration Groups (Fig. 6D).

**Fig. 7:**
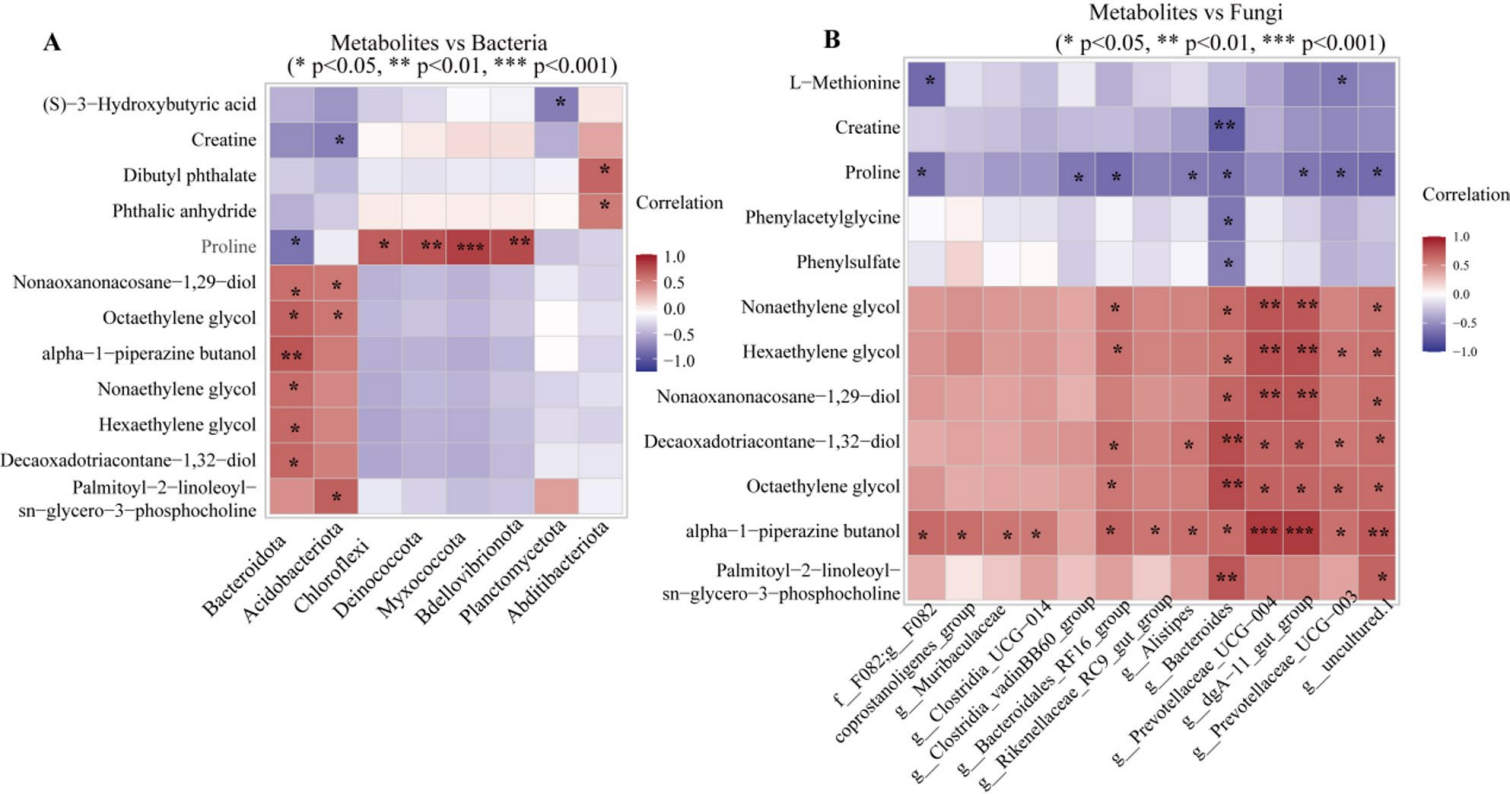
Correlation networks between microbial taxa and blood metabolites. (**A**) Correlation network between bacterial taxa and significantly altered blood metabolites (Spearman/Pearson, *p* < 0.05). (**B**) Correlation network between fungal taxa and blood metabolites. Red/blue edges indicate positive/negative correlations (|r| > 0.5, *p* < 0.05)

Pearson correlation analysis between top20 metabolites and microbial taxa (|r|>0.6, *p <* 0.05) revealed 5 strong positive bacterial correlations: Proline with *Myxococcota*, *Bdellovibrionota*, *Deinococcota* (*r* = 0.706–0.837); alpha-1-piperazine butanol with Bacteroidota (*r* = 0.743). Fungal communities exhibited 15 strong correlations: alpha-1-piperazine butanol strongly correlated with *Prevotellaceae_UCG-004* and dgA-11_gut_group (*r* = 0.858); polyethylene glycols and long-chain compounds correlated with *Bacteroides*, *Prevotellaceae_UCG-004*, and dgA-11_gut_group (*r* = 0.731–0.788); creatine showed strong negative correlation with *Bacteroides* (*r*=-0.756) (Fig. 7A-B).

### Correlations between hormones and cervical microbiota

**Fig. 8:**
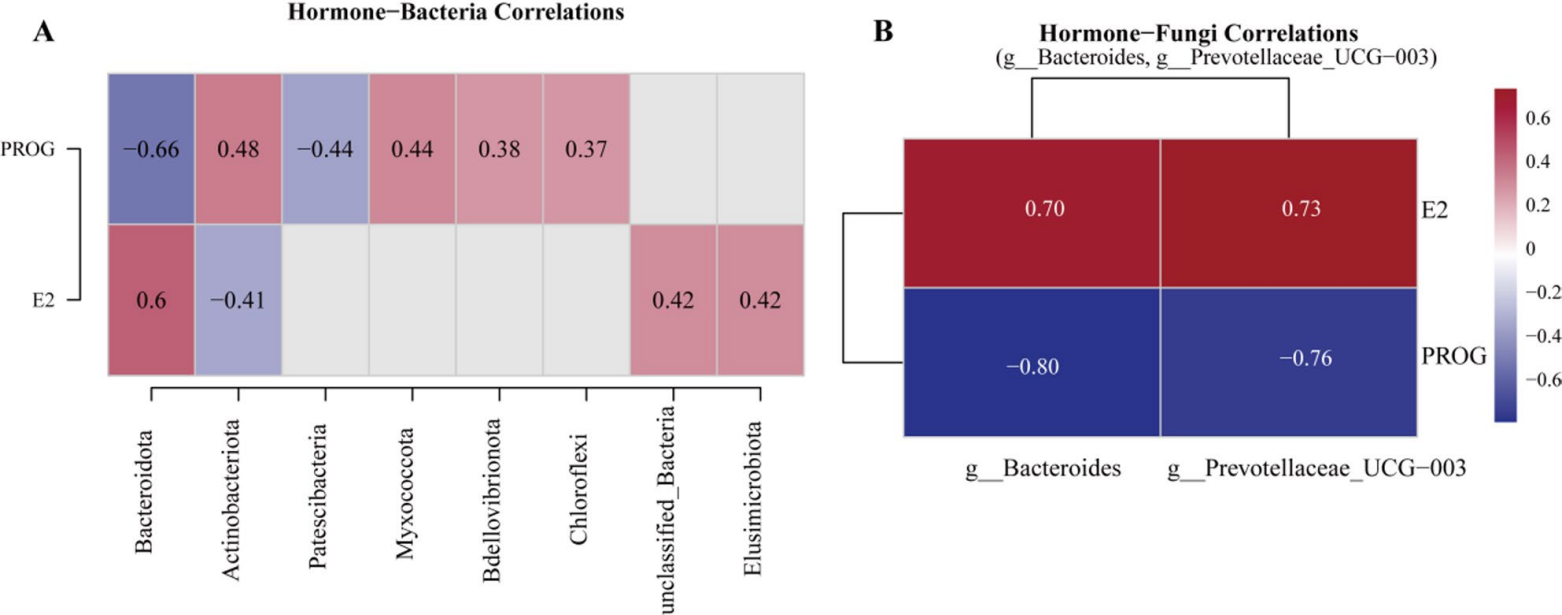
Correlations between reproductive hormones and cervical microbiota. (**A**) Heatmap showing Spearman correlations between serum hormone concentrations (E2, PROG) and relative abundances of bacterial phyla. (**B**) Correlations between hormones and fungal genera (*Bacteroides*, *Prevotellaceae*_UCG-003, *p* < 0.05)

Spearman correlation analysis revealed associations between reproductive hormones and cervical microbiota (Fig. 8). PROG was negatively correlated with *Bacteroidota* (*r* = -0.656) and positively with *Actinobacteriota* (*r* = 0.485) and Myxococcota (*r* = 0.438). E2 showed a positive correlation with *Bacteroidota* (*r* = 0.604) and a negative correlation with *Actinobacteriota* (*r* = -0.414) (Fig. 8A). At the fungal genus level, E2 was positively correlated with *Bacteroides* (*r* = 0.703) and *Prevotellaceae*_UCG-003 (*r* = 0.734), while PROG showed negative correlations with these genera (*r* = -0.796 and *r* = -0.756) (Fig. 8B).

## Discussion

This study revealed significant alterations in blood metabolic characteristics and cervical microbial communities in Simmental cows during estrus. Through ELISA analysis, we observed markedly increased serum concentrations of total TP, ALB, and GLU during the estrus phase, findings that are consistent with those of Wu et al.‘s research on dairy cattle. Notably, dairy cows exhibit substantial variations in serum biochemical parameters across different physiological stages, particularly during the estrus and lactation periods [13]. These changes may be associated with energy metabolism and reproductive status. Furthermore, this study revealed a significant increase in the GLB concentration during estrus in dairy cattle. This phenomenon may be related to immunological alterations during the estrus cycle, as globulins are components of the immune system [29]. During estrus, immune modulation may contribute to increased globulin concentrations and altered acute-phase protein (APP) levels [30]. These protein changes could serve as indicators for monitoring physiological status in cattle [30]. Together, these findings suggest that serum biochemical parameters during estrus may have implications for reproductive efficiency and health management in Simmental cattle production systems.

This study also revealed significant differences (*p* < 0.01) in the hormonal profiles between the estrus and nonestrus phases in Simmental cattle. Specifically, the estrus group presented markedly elevated levels of E2, IGF-1, and INH-B, coupled with significantly reduced PROG concentrations, a hormonal pattern characteristic of ruminant estrus cycles. These findings provide hormonal benchmarks for subsequent investigations into blood metabolomics and the cervical microbiome during estrus. The consistent patterns observed in hormonal and biochemical parameters suggest potential for developing estrus detection technologies, though broader validation across larger populations and diverse management systems is needed. From a research design perspective, these baseline data provide an essential physiological reference framework for subsequent metabolomic studies, which may facilitate more precise elucidation of metabolic pathway regulation during estrus [31].

Using UPLC‒MS/MS technology, we systematically analyzed the blood metabolic profiles of Simmental cattle during estrus and identified significant metabolic reprogramming associated with the estrus state. Pathway analysis revealed that these differentially abundant metabolites were enriched primarily in amino acid metabolism and lipid metabolism pathways. Within amino acid metabolism, the tryptophan, phenylalanine/tyrosine, and arginine/proline metabolic pathways exhibited particularly strong associations with both estrus behavior and uterine blood flow regulation in beef cattle. The tryptophan metabolic pathway is involved in neurotransmitter synthesis and has been linked to behavioral regulation in other species [32]. Phenylalanine and tyrosine are precursors to neurotransmitters, and their metabolic products may be relevant to estrus behavior [33]. Furthermore, arginine metabolism is connected to nitric oxide (NO) production, a key vasodilatory mediator that influences blood flow, including uterine vascular dynamics [33]. Proline metabolism participates in cellular osmoregulation and antioxidant capacity, functions that may support physiological stress responses. We note that while the enrichment of amino acid and lipid metabolism pathways in Simmental is broadly consistent with findings in Holstein dairy cows [11], the specific metabolites identified (e.g., prolylleucine, erucifoline N-oxide) may differ. Studies in sheep have also demonstrated breed-specific metabolic patterns across reproductive periods [34], suggesting that metabolic adaptations during estrus may vary by breed due to genetic background and production purpose (dairy vs. beef vs. dual-purpose).

During follicular development, lipid metabolism remodeling is particularly prominent. Previous studies have shown that alterations in sphingolipid metabolism, glycerophospholipid metabolism, and ether lipid metabolism are closely associated with follicular development mechanisms [35–37]. Sphingolipid metabolism has been linked to cell proliferation regulation [36], while glycerophospholipid metabolism may influence membrane fluidity and signaling pathways relevant to follicular development [37]. Ether lipids are involved in cellular signaling and antioxidant defense, and their metabolic changes may accompany follicular growth and maturation [35]. Investigating these metabolic pathways may provide insights into the biological basis of follicular development and inform strategies for improving estrus expression and fertility in beef cattle.

This study provides a comprehensive analysis of the cervical microbiome in Simmental cattle during estrus, revealing significant associations between estrus status and specific microbial community structures. This study revealed that the estrus state was associated with reduced microbial richness and lower overall diversity—a finding that may reflect unique ecological selection pressures within the cervical microenvironment during estrus [38]. The most notable finding was a 13.36% increase in the relative abundance of *Bacteroidetes* in the estrus group, which may have important physiological implications [39, 40]. Recent studies suggest that *Bacteroidetes* species can degrade mucopolysaccharides to produce short-chain fatty acids (SCFAs), metabolites known to modulate local immune environments [39, 40]. Particularly noteworthy is the identification through LEfSe analysis of characteristic estrus-associated microbial families, including *Prevotellaceae* and *Ruminococcaceae*, both of which are known for their potent polysaccharide-degrading capabilities [38, 41]. These findings suggest that cervical microbiome composition shifts during estrus, potentially reflecting adaptations in the local metabolic environment.

The enrichment of *Collinsella* in the characteristic microbiota of the nonestrus group may reflect luteal-phase metabolic patterns. This genus is associated with bile acid metabolism. Moreover, progesterone has been shown to modulate gut microbial composition through bile acid signaling pathways, which may contribute to epithelial barrier function [42]. These observations suggest *Collinsella* as a potential marker of nonestrus status, though further research is needed to establish this association [42].

Notably, the nonestrus group also showed enrichment of *Staphylococcus* and *Corynebacterium*, consistent with reports that low abundances of these genera before insemination improve pregnancy rates [21]. Comparisons with other breeds provide context. Similar phyla dominance was observed in Yanbian and Yanhuang cattle, with breed-specific beta diversity differences [43]. Uterine microbiota in Angus heifers also varied with puberty status [44]. Metabolically, our findings align with Holstein studies [11], while sheep data suggest breed-specific patterns [34], supporting the need for multi-breed investigations.

Through comprehensive analysis of the cervical mycobiota in Simmental cattle, this study systematically characterized the association between estrus and fungal community structure in the reproductive tract. The estrus state was associated with significantly lower α diversity indices (Chao1 and observed species), which may reflect unique ecological selection pressures during estrus. PCoA showed clear separation between the two groups, suggesting that estrus status is strongly associated with fungal community composition.

The estrus group presented an 11.18% greater relative abundance of *Ascomycota* (78.38% vs. 67.20%), whereas *Basidiomycota* was characteristically distributed in nonestrus samples (18.08%). This pattern aligns with reports that reproductive hormones may influence fungal community composition [45, 46]. Notably, the dominance of *Penicillium*, *Saccharomycopsis* in estrus may be physiologically significant, as this yeast genus metabolizes glycogen to produce lactate, which could contribute to a sperm-friendly weakly acidic environment [47]. Conversely, *Penicillium* in nonestrus samples may be associated with elevated PROG, and PROG has been associated with the growth of certain *Penicillium* strains, possibly contributing to luteal-phase immune homeostasis [48]. Estrus-characteristic taxa such as estrus andestrus are known to produce phytoestrogen-like compounds, suggesting potential interactions between fungal metabolism and host endocrine pathways [49–51]. Nonestrus-enriched *Mortierellomycota* have been associated with lipid metabolism in other contexts [52], though their role in the reproductive tract requires further investigation.

The correlation analysis revealed significant associations between specific microbial taxa and blood metabolites. The positive correlations between *Bacteroides* and bile acids align with the known role of this genus in bile acid metabolism [25], while associations between *Candida* and lipid metabolites suggest potential fungal contributions to host metabolism. *Melanocarpus* showed complex correlation patterns with multiple metabolites. These findings provide a foundation for understanding host-microbiota metabolic interactions during estrus, though functional experiments are needed to establish causality.

Correlation analysis revealed associations between reproductive hormones and cervical microbiota. E2 was positively correlated with Bacteroidota, while PROG showed a negative correlation with this phylum, consistent with the 13.36% increase in *Bacteroidetes* during estrus [53]. E2 was positively correlated with the fungal genera *Bacteroides* and *Prevotellaceae_UCG-003* (*r* > 0.7), whereas PROG showed negative correlations with these genera. These findings align with Poole et al. [53], who reported an increasing trend in Bacteroidota in the uterus of high PROG + low E2 cows, and Wu et al. [54], who demonstrated significant associations between reproductive hormones and gut microbiota. These results support the existence of a hormone-microbiota regulatory network in the reproductive tract, where E2 and PROG may modulate microbial communities to create a favorable environment for fertilization during estrus.

Several limitations of this study should be acknowledged. First, the sample size (*n* = 6 per group) is relatively modest, which may limit statistical power and the generalizability of the findings. Although the multi-omics approach and rigorous phenotyping provided robust intra-group characterization, we recognize that validation in larger, independent cohorts will be an important next step. Second, the cross-sectional design captures a single time point per animal and does not capture the dynamic changes that may occur across the estrus cycle within the same individual. Future longitudinal studies tracking cows throughout the cycle would help clarify the temporal relationships between metabolic shifts and microbial community restructuring during estrus. Third, the integrative analyses presented here are correlational in nature and do not imply causation. The observed associations between microbial taxa and metabolites should therefore be considered hypothesis-generating, and we view functional experiments—such as in vitro co-culture studies or targeted interventions—as essential directions for future research to test the mechanistic hypotheses raised by this work.

## Conclusion

In Simmental cows during estrus, serum biochemical parameters (TP, ALB, and GLU) and hormone levels (IGF-1 and 6PG) were significantly elevated, indicating increased protein and carbohydrate metabolic activities. Metabolomic analysis revealed 258 differentially abundant metabolites that were enriched primarily in amino acid and lipid metabolism pathways, suggesting that these metabolic pathways play crucial roles during estrus. Cervical microbiota analysis revealed that the bacterial community in estrus cows was dominated by *Firmicutes* and *Bacteroidetes*, whereas the fungal community was predominantly *Ascomycota*, with significantly reduced fungal diversity and richness. LEfSe analysis identified 22 bacterial biomarkers and 16 fungal biomarkers. This study provides novel insights into the metabolic and microbial characteristics of estrus in Simmental cattle, contributing to a deeper understanding of their reproductive physiological mechanisms.

## Supplementary Information

Below is the link to the electronic supplementary material.

Supplementary Material 1

Supplementary Material 2

## Data Availability

The sequencing data of microbial biomolecular markers are available in the NCBI database under accession number PRJNA1293676 (https://www.ncbi.nlm.nih.gov/bioproject/PRJNA1293676). The metabolome data are available in the MetaboLights database under accession number MTBLS12755 (https://www.ebi.ac.uk/metabolights/MTBLS12755).
