# Supplementary material for: Multi-omics characterization of blood metabolites and cervical microbiota associated with estrus in simmental cattle
Source: Anim Microbiome. 2026 Apr 15;8:47. doi: 10.1186/s42523-026-00571-8 (PMC13085721; doi:10.1186/s42523-026-00571-8)
Supplement: Supplementary file 1 — Supplementary Material 1 [file 42523_2026_571_MOESM1_ESM.docx]

****Supplementary Figure 1. Serum metabolomic analysis of estrous vs. nonestrous cattle.****


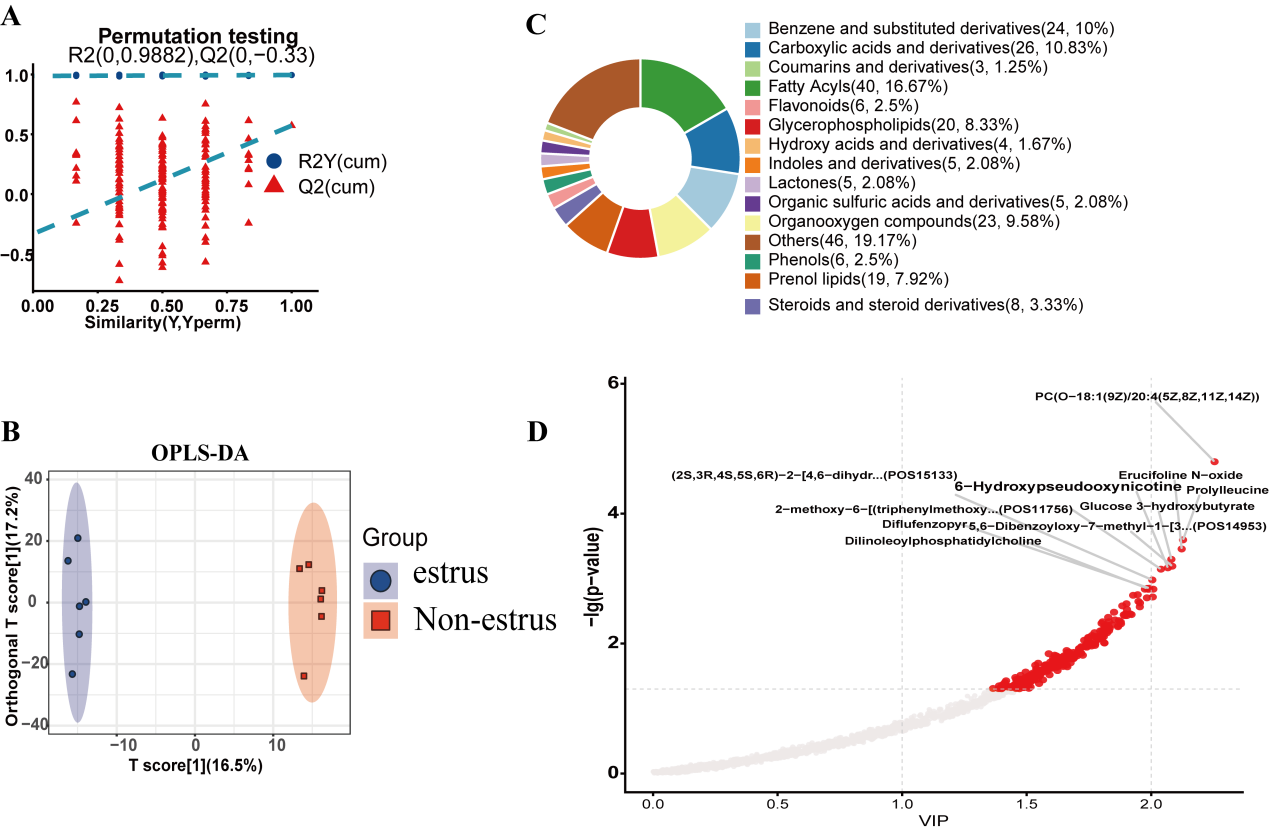


(A) Permutation test. (B) OPLS-DA. (C) Circular plot of metabolite classification (HMDB class). (D) Scatter plot of VIP scores vs. p values.
