# Supplementary material for: Multi-omics characterization of blood metabolites and cervical microbiota associated with estrus in simmental cattle
Source: Anim Microbiome. 2026 Apr 15;8:47. doi: 10.1186/s42523-026-00571-8 (PMC13085721; doi:10.1186/s42523-026-00571-8)
Supplement: Supplementary file 2 — Supplementary Material 2 [file 42523_2026_571_MOESM2_ESM.docx]

**Supplementary tables**

Supplementary table 1: Cow Basic Information and Estrus Signs

| Cow ID | Age (years) | Parity | Weight (kg) | Body Condition Score (1-5) | Dominant Follicle (mm) | Estrus Behavior Score |
| --- | --- | --- | --- | --- | --- | --- |
| E-01 | 4.5 | 2 | 655 | 3.2 | 10.2 | 3 |
| E-02 | 4.3 | 2 | 630 | 3.1 | 8.5 | 2 |
| E-03 | 4 | 2 | 670 | 3.3 | 9.8 | 3 |
| E-04 | 4 | 2 | 645 | 3 | 11 | 3 |
| E-05 | 4.3 | 2 | 660 | 3.4 | 9.2 | 3 |
| E-06 | 4.2 | 2 | 654 | 3.2 | 8.9 | 3 |
| NE-01 | 4.2 | 2 | 620 | 3 | - | 0 |
| NE-02 | 4.2 | 2 | 635 | 3.2 | - | 0 |
| NE-03 | 4.3 | 2 | 650 | 3.1 | - | 0 |
| NE-04 | 4.1 | 2 | 625 | 3.3 | - | 0 |
| NE-05 | 4.4 | 2 | 640 | 3.2 | - | 0 |
| NE-06 | 4.2 | 2 | 656 | 3.2 | - | 0 |

Supplementary table 2: Hormonal and Ovarian Characteristics

| Cow ID | Vaginal Mucus Crystallization | Progesterone (ng/mL) | Ovarian Ultrasound Features | Sampling Stage |
| --- | --- | --- | --- | --- |
| E-01 | Fern-like | 0.6 | Single DF (10.2mm) | At estrus signs |
| E-02 | Fern-like | 0.5 | 2 SF (6.5, 8.5mm) | At estrus signs |
| E-03 | Fern-like | 0.8 | Single DF (9.8mm) | At estrus signs |
| E-04 | Fern-like | 0.7 | Single DF (11.0mm) | At estrus signs |
| E-05 | Fern-like | 0.5 | Single DF (9.2mm) | At estrus signs |
| E-06 | Fern-like | 0.7 | Single DF (9.6mm) | At estrus signs |
| NE-01 | No | 2.9 | CL present | 10 days post-estrus (diestrus) |
| NE-02 | No | 3.1 | CL present | 10 days post-estrus (diestrus) |
| NE-03 | No | 3.5 | CL present | 10 days post-estrus (diestrus) |
| NE-04 | No | 2.9 | CL present | 10 days post-estrus (diestrus) |
| NE-05 | No | 3.3 | CL present | 10 days post-estrus (diestrus) |
| NE-06 | No | 3.5 | CL present | 10 days post-estrus (diestrus) |

Supplementary table 3: Estrus Behavior Scoring System (Detailed Criteria)

| Score | Description (Behavioral Signs) |
| --- | --- |
| 0 | No estrus signs – No mounting, no vulvar swelling, no mucus discharge. |
| 1 | Weak signs – Slight restlessness, minimal mucus discharge, no standing heat. |
| 2 | Moderate signs – Clear mucus discharge, vulvar swelling, occasional mounting but no standing heat. |
| 3 | Strong signs – Frequent mounting, standing heat (accepts mounting), copious clear mucus, vulvar swelling. |
